# Put you in the problem: Effects of self-pronouns on mathematical problem-solving

**DOI:** 10.1177/17470218231174229

**Published:** 2023-05-23

**Authors:** Sheila J Cunningham, Zahra Ahmed, Joshua March, Karen Golden, Charlotte Wilks, Josephine Ross, Janet F McLean

**Affiliations:** 1 School of Applied Sciences, Abertay University, Dundee, UK; 2 Psychology, School of Humanities, Social Sciences and Law, University of Dundee, Dundee, UK

**Keywords:** Self, self-referencing, problem-solving, attention, numeracy, development

## Abstract

Self-cues such as personal pronouns are known to elicit processing biases, such as attention capture and prioritisation in working memory. This may impact the performance of tasks that have a high attentional load like mathematical problem-solving. Here, we compared the speed and accuracy with which children solved numerical problems that included either the self-cue “you,” or a different character name. First, we piloted a self-referencing manipulation with *N* = 52, 7 to 11 year-olds, testing performance on addition and subtraction problems that had either a single referent (“You”/“Sam”) or more than one referent. We took into account operation and positioning of the pronoun and also measured performance on attention and working memory tasks. We found a robust accuracy advantage for problems that included “you,” regardless of how many characters were included. The accuracy advantage for problems with a self-pronoun was not statistically associated with individual differences in attention or working memory. In our main study (9 to 11 year-olds, *N* = 144), we manipulated problem difficulty by creating consistently and inconsistently worded addition and subtraction problems. We found significantly higher speed and accuracy for problems that included “you.” However, this effect varied by task difficulty, with the self-pronoun effect being strongest in the most difficult inconsistently worded, subtraction problems. The advantage of problems with a self-pronoun was not associated with individual differences in working memory. These findings suggest that self-cues like the pronoun “you” can be usefully applied in numerical processing tasks, an effect that may be attributable to the effects of self-cues on attention.

Self-processing biases in cognition are well established in both children and adults, evidenced by a robust tendency to preferentially perceive, attend to, and remember information relating to self more than information relating to other people or non-social cues (Humphreys & Sui, 2016; Rogers et al., 1977; Symons & Johnson, 1997). Extensive research on “own-name” and “own face” effects has shown that these self-cues capture attention when presented as stimuli (Tong & Nakayama, 1999; Yang et al., 2013), even when this is detrimental to concurrent task performance (Bargh & Pratto, 1986; Brédart et al., 2006; Röer et al., 2013; Yin et al., 2019). However, when self-cues are integrated within a task, they can also enhance task engagement and processing (Turk et al., 2015). For example, self-pronouns such as “you” (to the participant) or “I” (from the participant’s perspective) can make written narratives more personally resonant (see Brunyé et al., 2009; Orvell et al., 2020). There may therefore be practical benefits to self-processing biases: if self-cues reliably attract attention and engagement, this could facilitate performance on tasks with high processing requirements such as mathematical problem-solving. For example, in a problem that starts “If you have three apples, and I take away two. . .” the natural tendency to simulate “you” from a personal perspective might facilitate your ability to compute how many apples are left.

Surprisingly, little empirical attention has been paid to this prediction, although there is some preliminary evidence that self-pronouns can impact aspects of mathematical processing (D’Ailly et al., 1995, 1997). D’Ailly et al. (1995) examined linear ordering problems in which participants read a story containing order information (e.g., *“Students are voting for their leader. Tom gets more votes than John. John gets more votes than You. You get more votes than Rod, and Rod gets more votes than Paul”*) and were then questioned on specific comparisons (e.g., *did Rod get more votes than Tom?*). For some participants, the list included the self-pronoun “you” either in an anchoring position (i.e., first or last in the list) or one of the middle positions, while other participants read lists with no self-pronouns. Across three experiments with young adults, response time (RT) data showed an advantage for self-referenced problems, but only when “you” was positioned in an anchoring position. When “you” was in the middle of the list RT increased, suggesting it distracted participants from the processing task in this position. The authors concluded that the role of the self was to anchor the order of the listed information, although the cognitive mechanisms that might underpin this role were not expounded.

The cognitive mechanisms responsible for producing the effects of self-pronouns reported by D’Ailly et al. are likely to derive from the effects of self-cues on attention and relatedly, working memory. Working memory is the system that holds information temporarily while it is processed, whereas attention can be defined as either the limited capacity resource that can be directed to storage and processing or the control process that determines the allocation of this attentional resource (i.e., executive attention; see Cowan, 2017; Oberauer, 2019). In terms of problem-solving, working memory is required to temporarily store the information made active by the problem (e.g., referents, orders), allowing this information to be processed accurately. However, if processing is complex and therefore requires a high level of attentional resources, it may compete with the resources required to concurrently store the active information, particularly over very short periods (Cowan, 2005). Thus, if there is a high degree of processing required then some active information may be lost, resulting in errors. Errors can also be caused by a high storage demand due to an overload of information, which reduces the capacity for accurate processing. Finally, when there is competition for attentional resources, the executive attention system must allocate resources between tasks, a process that in itself reduces attentional resource availability by demanding cognitive control (Oberauer, 2019).

Importantly, the self could play a role at multiple levels of the attention system. First, it is possible that self-associated information could be more efficiently stored, as self-referenced items are associated with enhanced organisation (Klein & Kihlstrom, 1986; Klein & Loftus, 1988) which may operate even within a short-term working memory capacity (see Yin et al., 2019). Furthermore, the self is not a new character whose name needs to be remembered and tracked during processing. This should reduce storage requirements, so including self-pronouns in tasks such as mathematics word problems may reduce their working memory load. Second, it is possible that self-cues increase processing efficiency, by ensuring that attentional resources are directed automatically to the current task rather than being directed elsewhere or consciously focussed on the task (see Humphreys & Sui, 2016). Self-cues may therefore reduce the executive attentional requirements of the task, freeing greater capacity for storage and processing. Tasks with a high working memory load (especially verbal working memory, which is harder to maintain) lead to vulnerability to distraction, as there are insufficient attentional resources to allow conscious control over attention allocation (Kelley & Lavie, 2011; Lavie et al., 2004). By reducing the need for conscious attention allocation, self-cues circumvent this executive requirement and therefore minimise the overall attentional load of the task (cf. Lavie, 2010).

A combination of these attentional processes may explain why the position of the self-cue influences its effects on task performance. If a self-cue is presented in an anchoring position at the beginning of a problem or list (Jensen, 1962), then it is not distracting because it elicits automatic attention allocation to the relevant information without executive control. However, if presented in the middle of a problem, it may replace the original anchor point and distract from the storage and processing of the other relevant information. Self-cues are distracting when presented alongside other target cues (e.g., in visual search or auditory monitoring tasks; Brédart et al., 2006; Röer et al., 2013) as they attract attention and disrupt the processing of other cues. When presented in the middle of a long list, the distracting effect of “you” may therefore outweigh the benefits of automatic attention capture or having fewer referents to hold in working memory. D’Ailly et al.’s (1995) findings can therefore be accounted for by the interaction of self-cues’ facilitating and distracting effects.

Complicating this explanation are findings from a second study by D’Ailly and colleagues, in which the difficulty as well as position of cues was manipulated. D’Ailly et al. (1997) presented word problems orally to 7- to 11-year-old children, half of which contained the self-pronoun “you” instead of another referent name. Two types of problem were presented, varying by difficulty: “Compare-Unknown” questions, in which the information required to solve the problem is relatively easy to extract (e.g., “*John has 5 marbles. Peter has 2 marbles more than John. How many marbles does Peter have?*”) and “Referent-Unknown” problems, which add working memory load to the problem by requiring the participant to transfer information about one referent to the other to answer the question (e.g., “*John has 5 marbles. John has 2 marbles more than Peter. How many marbles does Peter have?*). In referent unknown questions, the difficulty is increased by the terminology not being congruent with the required arithmetic operation (e.g., when the question includes the phrase ‘more than,” the operation required is subtraction; Lewis & Mayer, 1987). Similar to D’Ailly et al. (1995), the position of the “you” referent within the word problem was manipulated. D’Ailly et al. found that children requested fewer repeats and had more accurate and fast responses for questions with a self-pronoun, although this pattern was complicated by question difficulty. In the easier Compare-Unknown questions, self-pronouns improved accuracy and RT whether they were in the first or second position in the problem (i.e., “you” as the referent with the known quantity, or “you” as the unknown referent). However, in the more difficult Referent-Unknown problems, the effect of self interacted with pronoun position. Self-pronouns only improved accuracy when the “you” was the first, known quantity; when “you” was in the second, unknown quantity this had a negative effect on performance. Response time data revealed a similar interaction; in referent-unknown problems, the inclusion of self-pronouns did not affect RT when “you” was in the first, known position, but increased RT when it was in the second, unknown position. D’Ailly et al.’s (1997) findings suggest that when task difficulty (and consequently, working memory load) is low, self-pronouns can have a beneficial effect regardless of position, but under more difficult processing conditions, self-pronouns can be disruptive if not in an anchoring role.

However, an issue with the interpretation of difficulty in D’Ailly et al.’s (1997) study is that while both addition and subtraction questions were included as trials, the reported results do not distinguish between the two. This is important because these two operations differ in their working memory requirements (see Raghubar et al., 2010). In non-experts whose responses are not retrieval-based, addition involves counting on (forward) so has a lower working memory load than subtraction, which typically involves the more difficult counting backward technique (i.e., “‘taking away”; Baroody, 1984; Hopkins et al., 2020; although see Daroczy et al., 2015 for more detailed analysis of linguistic and computation complexity). Furthermore, when children acquire an understanding of “indirect addition,” whereby subtraction is an inversion of the addition process (e.g., taking 5 from 8 to leave 3 is the inverse of adding 3 and 5 to get 8), they may use addition first and then apply inversion. This would again increase the working memory load of subtraction problems (Thompson, 1999). If the impact of self-cues on problem solving varies by problem’s working memory load, it may therefore vary across operations.

As well as utilising self-cues, the link between self-relevance and working memory in educational tasks has been examined from the “personalization” perspective. Personalization involves educators adapting activities to suit individual children’s personal hobbies and interests, such as creating a football-based mathematics quiz for a child who likes football (for review see Reber et al., 2018). This approach tends to elicit additional task engagement and motivation in students, although its effects on performance are more mixed (e.g., see Akinsola & Awofala, 2009; Bates & Wiest, 2004; Høgheim & Reber, 2015; Van de Weijer-Bergsma & Van der Ven, 2021), perhaps partly due to variation in how closely the intervention matches learners’ unique prior experiences (Walkington & Bernacki, 2017). Van de Weijer-Bergsma and Van der Ven (2021) assessed the effect of personalization on students’ perceived cognitive load, predicting that when the contextual information within a problem is familiar, then the working memory requirements may be reduced, releasing more resources for problem-solving. However, in testing, neither self-reported cognitive load nor performance was found to be affected by a personalization intervention, suggesting that this approach may not be sufficiently reliable across children. Personalization also engenders the issue of having to research and design personal materials for every individual child. In contrast, using personal pronouns provides a tool that is applicable to every child, so may be more suitable for testing in a school context.

The current study was designed to explore the effects of self-cues on problem solving in school children. First, we piloted the self-referencing manipulation on 7- to-11-year-old children to provide a conceptual replication of D’Ailly and colleagues’ (1995, 1997) findings and establish whether self-cues reliably impact performance in our mathematical problem-solving task. Self-cues were expected to free attentional resources for problem solving by facilitating working memory storage (through increased organisation, familiarity, and a reduced need to keep multiple novel characters active) and automatically capturing attention. Problems that included the self-cue “you” were therefore expected to be solved with greater accuracy and speed than those without a self-cue. Following the pilot, our main study focussed on establishing whether operation and problem difficulty impact the self-reference effect, such that the automatic attention-grabbing properties of the self may be facilitative for easy problems, but disruptive when task difficulty is high. We also assessed the effects of individual differences in attention and working memory capacity on any accuracy or RT advantage for self-referent problems. Children who have lower working memory capacity may benefit more from conditions that reduce the attentional load of the problem relative to children who have greater capacity (see Miller et al., 2006; Van Gerven et al., 2003). Furthermore, children who are more able to sustain attentional focus or switch attention to a task without the aid of self-cues may benefit less from the attention capture these cues provide (see Schwaighofer et al., 2016). This work is critical to answer key questions about the practical application of the self-referencing manipulation in the classroom, determining under which conditions self-referent framing of numerical problems may aid problem processing, and for whom.

## Pilot study

Our pilot study was primarily designed to establish the impact of including the personal pronoun “you” on children’s performance on a word problem-solving task. The main hypothesis was therefore that children would be faster and more accurate on problems that included a self-referent pronoun than those that did not (D’Ailly et al., 1997). Manipulation of additional factors allowed us to determine whether these should be included in the main study. First, to test the suggestion that self-pronouns may function by reducing the number of characters the child has to hold in mind, we varied the number of referents included in the problem (single versus multiple). If self-reference effects are based solely on reducing the working memory load of holding multiple novel referents active, they should be strongest in multiple referent conditions. On the contrary, if self-reference effects operate by enhancing attention, they should be present regardless of referent number. We manipulated the operation required to solve the problem (i.e., addition or subtraction) and the position of the self- or other-referent term, positioning it as either the first anchoring word or in a later non-anchoring position. Based on D’Ailly et al.’s (1997) finding that the self may support attention and problem processing in an anchoring position, but be disruptive when presented later in the problem, particularly for more difficult tasks, we expected that the effect of referent may be stronger when self is in the anchoring position than when it is in a non-anchoring position, especially in subtraction problems. Given that the proposed mechanisms to support any effect of self-pronouns on children’s problem solving are rooted in attentional capture and capacity, the pilot study also included measures of children’s attentional processing. Children who have lower working memory capacity may benefit more from conditions that reduce the attentional load of the problem relative to children who have greater capacity (see Miller et al., 2006; Van Gerven et al., 2003). Furthermore, children who are more able to sustain attentional focus or switch attention to a task without the aid of self-cues, may benefit less from the attention capture they provide (see Schwaighofer et al., 2016). Therefore, measures of working memory, sustained and selective attention, and attention switching were included in the pilot study.

### Method

#### Participants and design

A total of 52 children completed the study, comprising eleven 7 year-olds (45% male, age range 84 – 93 months), twenty-four 8 year-olds (46% male, age range 96–107 months), and seventeen 9 year-olds (53% male, age range 108–119 months). All participants were Primary 4–5 pupils at a local primary school and had no reported problems speaking or reading English. The children were tested with the written consent of a parent or guardian and provided verbal assent, and the research was approved by Abertay University’s Research Ethics Committee.

The pilot had a repeated-measures design of 2 (Referent: Self, Other) × 2 (Operation: Addition, Subtraction) × 2 (Tracking: Multiple referents, Single referent) × 2 (Position: Anchoring, Not anchoring). Dependent measures were problem-solving accuracy and RT. Participants’ performance on the attentional measures was also included for exploratory analysis. Based on the very large effect size (*η_p_*^2^ = .31) reported for the only similar extant experimental finding (D’Ailly et al., 1997: the main effect of self-pronoun on mathematics accuracy), an appropriate sample size of 50 participants was calculated by G*Power 3.1.9.7 (α = .05, power = .95). However, it should be noted that this power calculation focussed on replication of the self-reference effect rather than interactions, and interpretation of operation, tracking and position interactions in the pilot study should therefore be treated with caution.

#### Materials and procedure

Children were tested individually by an experimenter, seated at a table in a quiet area of their school. Participants first completed the attentional measures, comprising forward and backward digit span (BDS), and three subtests from the TEA-Ch (Manly et al., 1999). In the forward digit span test, the experimenter read out aloud a sequence of numbers at a pace of one per second, and asked the participant to repeat them verbally. Sequences started at two digits and increased to a maximum of nine. There were two trials at each sequence length; if a child failed both trials then the test was terminated. Next, the BDS test was administered, following the same procedure as the forward test but with the participant asked to verbalise the sequence in reverse order.

After the digit span tasks, each child completed the three TEA-Ch tasks, beginning with the measure of selective attention, *Map Mission*. Participants were presented with a laminated city map and instructed to circle as many of the target symbol (a knife and fork “restaurant” symbol) as possible in one minute, with the experimenter providing a verbal start and stop signal. Participants then completed the measure of attention switching, *Opposite Worlds*. For this task, the participant was shown a pathway of linked boxes, each containing the digits “1” or “2.” They were asked to complete the first two pathways (“Same World”) by reading the digits aloud as they appeared in the boxes. They then completed two “Opposite Worlds” pathways, in which they were instructed to say the opposite digit (e.g., say “2” when there is a “1” in the box). Finally, children completed the test of sustained attention task *Score*. Wearing headphones, participants listened to a series of laser sounds presented on a CD across 10 trials. Children were instructed to keep track of the number of laser sounds played, as they would if keeping score during a video game. Participants were informed not to use their fingers to keep track, and to report the number verbally to the experimenter after each trial. Within each trial, the sounds were presented at irregular intervals to assess participants’ ability to sustain their attention.

Participants then completed the problem-solving task on a laptop, with experimental materials presented using E-Prime 2.0 software (Psychology Software Tools, 2002). Children were informed that they would complete a mathematics game in which they would be asked to solve some “sums” (i.e., mathematics problems). Participants were asked to read each question carefully and type their answer using the laptop keyboard, pressing the spacebar to submit the response. An example question was presented for practice. Once the participant confirmed they understood the question and completed the practice trial without any issues, the test began, with the experimenter present throughout.

Participants completed two blocks of self-paced trials, with the opportunity for a short break between blocks. The two blocks varied by Tracking condition, and each consisted of 24 problems, with three items per combination of manipulations. In the Multiple Referents block, each problem referred to two characters and one object (e.g., “*Sam has 3 stickers and Jack has 9 stickers. How many stickers do they have altogether?*”; see the Online Supplementary Materials for a full list of problems). Under the Single Referent condition, each problem referred to one character and two objects (e.g., *“Sam has 3 stickers and also has 5 cards. How many items does he have altogether?”*). Block order was counterbalanced across participants.

Within each block, half of the questions were Addition and half Subtraction problems, presented in an order randomised within block by the experimental programme. For addition problems, the sums comprised two positive integers and totals ranged from 5 to 15 (avoiding duplicate integers within sum and duplicate answers across trials), with half of the sums listing the larger integer first. The subtraction problems were created by reversing each of the addition sums, presenting the sum total as the first number in the subtraction problem followed by either the larger (50%) or smaller (50%) of the other two integers to take away.

For each participant, half of the questions under each Tracking and Operation condition were Self-referent (i.e., included a self-pronoun: e.g., *You had 5 grapes but Zahra took 2. How many grapes do you have now?’*), and half were Other-referent (i.e., only included other-referents: e.g., *“Sam had 5 grapes but Zahra took 2. How many grapes does Sam have now?”*). To transform a Self-referent problem into an Other-referent problem, the word “you” was replaced with the unisex three-letter name Sam, so that the other-referent name was presented the same number of times as the word “you” across the task. Other-referent pronouns were also manipulated to reduce self-projection, such that male participants were presented with female pronouns for Sam and female participants with male pronouns. The referent term “you” or “Sam” was presented as the anchoring first word in half of the problems, and as a subsequent (non-anchoring) word in the remainder. The inclusion of each addition and subtraction problem under either the Self-referent or Other-referent condition was counterbalanced across participants. Problem word lengths were matched exactly across Addition and Subtraction problems, and across Self- and Other-referent conditions. In total, completion of the attentional measures and problem-solving task took approximately 40–50 min per child.

### Data analysis

Participants’ performance on the problem-solving task was scored for accuracy (proportion of problems correctly answered in each condition) and RT (latency from question onset to response submission on correct trials). Due to experimenter error, 8 trials were presented with the wrong pronoun, and were excluded from the analyses. All data were coded automatically by E-Prime with the exception of one item for which the underlying mathematical problem 9–2, had been incorrectly replaced with 7–2 in the Multiple Referents version. These questions were re-scored manually so that if a child had answered 5, it was counted as a correct response. Exclusions were applied to the data. First, four individual trials with an RT below 200 ms (i.e., indicating accidental keypress) were removed. Participants were then removed if they were unable to process the numeracy questions effectively, as indicated by a mean accuracy below the sample mean minus 2.5 SDs (*n* = 1), or a mean RT greater than the mean plus 2.5 SDs (*n* = 1). These exclusions resulted in a total of 50 participants and 2,398 trials being included in the analyses.

Three separate analyses were planned: first on accuracy scores on the problem-solving task, then RT on the same task, and finally, on the relationships between individual differences (age, performance on working memory tasks) and any accuracy or RT advantage for items under the self-referent condition over other-referent items. Repeated measures ANOVAs were used to analyse accuracy and RT. All possible interaction terms were included in the analyses as a matter of transparency; those involving Referent are included in “Results” section to explore whether any effects of referent may be conditional on operation, the number of referents to be tracked, or referent position. Those that were not of primary interest (i.e., do not relate to Referent) are reported in the Online Supplementary Materials.

Three-way interactions were examined using two-way interaction analyses, and to follow up two-way interactions, simple main effects on Referent were conducted. Partial eta squared is reported as a measure of effect size, and by convention .01, .06, and .14 are considered small, medium, and large effects, respectively (Cohen, 1969; Richardson, 2011). Bivariate Pearson correlation was used to examine the strength and direction of relations between the continuous variables of self-advantage in accuracy and RT, and individual differences in age and working memory.

Data collection for the pilot study was preregistered as part of a PhD project. The PhD study preregistration is available at https://aspredicted.org/blind.php?x=YFL_WKB. The dataset and analysis scripts can be accessed at https://osf.io/naqz5/.

### Results

Performance was high overall, with a mean percentage accuracy of 87.9%, 95% CI [86.4%, 89.4%], and a mean RT of 16.0 s, 95% CI [15.4, 16.7] per accurate response.

#### Accuracy

Accuracy data (i.e., proportion of correct responses in each condition) were submitted to a 2 (Referent: Self, Other) × 2 (Operation: Addition, Subtraction) × 2 (Tracking: Multiple referents, Single referent) × 2 (Position: Anchoring, Not anchoring) within-participants ANOVA (see the Online Supplementary Materials for full mean and standard deviations broken down by condition). As Table 1 shows, the ANOVA revealed a main effect of Referent, with higher accuracy on trials with Self pronouns, *M* = 0.90, 95% CI [0.86, 0.93], than Other pronouns, *M* = 0.86, 95% CI [0.83, 0.90]. There was also a main effect of Operation, with more correct responses on Addition problems, *M* = 0.92, 95% CI [0.88, 0.96], than Subtraction problems, *M* = 0.84, 95% CI [0.80, 0.87].

**Table 1.** ANOVA for pilot study accuracy (all df_num = 1, df_den = 49).

Predictor	*F*	*p*	*η*_p_^2^
Referent	6.66	.013*	.120
Operation	20.89	<.001***	.299
Tracking	1.18	.282	.024
Position	3.13	.083	.060
Referent × Operation	0.00	.973	<.001
Referent × Tracking	3.56	.065	.068
Referent × Position	0.01	.913	<.001
Operation × Tracking	5.33	.025*	.098
Operation × Position	0.02	.896	<.001
Tracking × Position	5.02	.030*	.093
Referent × Operation × Tracking	0.03	.869	<.001
Referent × Operation × Position	4.98	.030*	.092
Referent × Tracking × Position	2.31	.135	.045
Operation × Tracking × Position	3.08	.085	.059
Referent ×Operation × Tracking × Position	1.25	.270	.025

* *p* < .05, ***p < .001.

**Figure 1.**
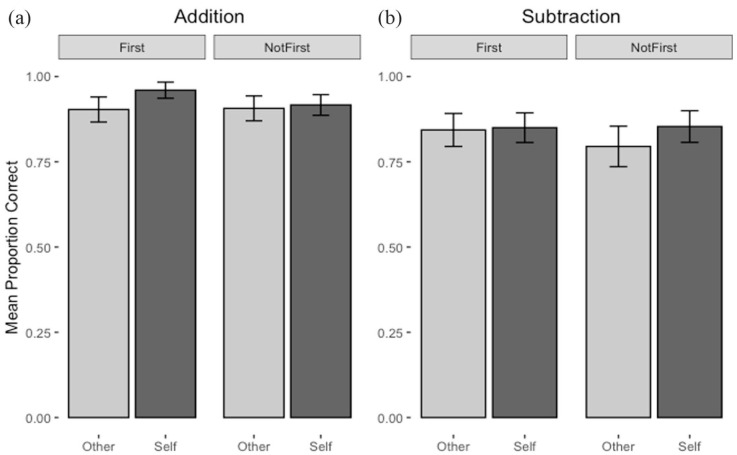
Mean proportion correct by referent and position for (a) addition and (b) subtraction in the pilot study. The error bars represent 95% confidence intervals.

The full list of interaction effects can be seen in Table 1, but we confine our report of paired comparisons to those of theoretical interest (i.e., those involving the Referent factor; see the Online Supplementary Materials for a full examination of other interaction effects). There was no Referent × Operation, Referent × Position interaction, or Referent × Tracking interaction. However, there was a three-way interaction between Referent × Operation × Position (see Figure 1). Analysis of interaction effects for Referent × Operation at each level of Position showed that the interaction was not significant when the referent was in the Anchoring position, *F*(1, 49) = 3.53, *p* = .07, *η_p_*^2^ = .07, nor when the referent was in the Not Anchoring Position, *F*(1, 49) = 1.64, *p* = .21, *η_p_*^2^ = .03. However, as the significance levels suggested some uncertainty, and to explore why the three-way interaction occurred, we conducted further simple main effects for Referent at each level of Operation and Position. These showed that the accuracy advantage for trials with a self pronoun over those without was significant in Addition problems when the repeated referent was in the anchoring position, *F*(1, 49) = 11.23, *p* = .002, *η_p_*^2^ = .19. A trend towards the opposite pattern for Subtraction problems (i.e., benefit of self-pronoun when the repeated referent was not in the anchoring position) was not significant, *F*(1, 49) = 3.14, *p* = .08, *η_p_*^2^ = .06.

#### Response time

We conducted the same 2 (Referent: Self, Other) × 2 (Operation: Addition, Subtraction) × 2 (Tracking: Multiple referents, Single referent) × 2 (Position: Anchoring, Not anchoring) repeated measures ANOVA on participants’ mean RT on correct responses (see Table 2 for ANOVA output).

**Table 2.** ANOVA for pilot study response time (all df_num = 1, df_den = 49).

Predictor	*F*	*p*	*η*_p_^2^
Referent	0.05	.826	<.001
Operation	111.56	<.001***	.695
Tracking	1.03	.315	.021
Position	6.58	.013*	.118
Referent × Operation	6.92	.011*	.124
Referent × Tracking	0.37	.545	.004
Referent × Position	0.08	.785	.002
Operation × Tracking	4.68	.035*	.087
Operation × Position	0.50	.484	.010
Tracking × Position	1.55	.219	.031
Referent × Operation × Tracking	0.54	.467	.011
Referent × Operation × Position	0.36	.553	.007
Referent × Tracking × Position	0.68	.414	.014
Operation × Tracking × Position	0.11	.741	.002
Referent × Operation × Tracking × Position	0.01	.905	< 001

* *p* < .05, ***p < .001.

There were main effects of Operation and Position, with shorter response latencies for addition problems, *M* = 13.5 s, 95% CI [11.5, 15.4] than subtraction problems, *M* = 18.6 s, 95% CI [16.7, 20.6], and for those in which the repeated referent (You/Sam) was positioned in the anchoring position, *M* = 15.7 s, 95% CI [13.8, 17.6] rather than later in the problem, *M* = 16.3 s, 95% CI [14.4, 18.3]. There was no main effect of Referent on RT, but Referent did interact significantly with Operation. Pairwise comparisons revealed that Addition problem responses were marginally faster with Self, *M* = 12.9 s, 95% CI [10.9, 15.0], than Other referents, *M* = 14.0 s, 95% CI [11.9, 16.0]; *t*(97.9) = 1.985, *p* = .0499. However, this was not the case for Subtraction problems where there were no RT differences between Self, *M* = 19.1 s, 95% CI [17.0, 21.1], and Other referent problems, *M* = 18.2 s, 95% CI [16.2, 20.2]; *t*(97.9) = −1.67, *p* = .10.

## Exploratory analysis: self-advantage associations

To explore relationships between the effects of self and measures of attention, a self-advantage score was calculated for both accuracy (performance under Self minus Other referent condition) and RT (performance under Other minus Self-referent condition). Raw scores were used for each of the attention measures (see Table 3), comprising maximum span in the forward digit span and BDS, total RT to the two Opposite Worlds in the *Opposite Worlds* task, number of items found in the *Map Mission* task and mean accuracy in the *Score!* task. As Table 3 shows, self-advantage for accuracy did not correlate significantly with age (in months) or any measure of sustained attention, attention switching, or attentional capacity (BDS). There was a significant negative correlation between the self-advantage in accuracy and total accuracy, *r*(48) = −.43, *p* = .002, suggesting that the self-advantage was larger for children who performed more poorly on the task overall. However, this is likely to be constrained by ceiling effects, such that children who do better on the tasks for self and other have less scope to show a self-reference advantage. The self-advantage in RT correlated positively with only one measure of sustained attention, the *Score!* task, *r*(48) = .40, *p* = .003, with children who had higher sustained attention scores more likely to show an RT advantage in self-referent trials. There was also a positive relationship between participants’ total accuracy of the numeracy task and their digit span, but no other correlations with self-advantage scores approached significance.

**Table 3.** Correlation matrix showing self-advantage scores and performance on the measures of working memory, sustained attention, and attention switching in the pilot study (all measures *N* = 50).

	*M*	*SD*	Pearson’s correlation coefficient							
			1	2	3	4	5	6	7	8
1. Self-advantage accuracy	0.03	0.09	—							
2. Self-advantage RT	0.03s	2.61s	−0.26	—						
3. Age in months	102.00	9.96	0.11	−0.18	—					
4. Total accuracy	0.87	0.12	−0.43**	0.17	0.01	—				
5. Forward digit span	8.48	2.04	−0.11	0.14	0.07	0.48*	—			
6. Backward digit span	4.52	1.90	−0.07	0.10	0.21	0.30	0.32*	—		
7. Attention switching (*Opposite World* task)	61.68	207.83	0.02	0.14	0.13	0.07	−0.04	−0.12	—	
8. Selective attention (*Map Mission* task)	25.38	7.34	0.07	−0.17	0.46***	0.01	0.09	−0.04	0.14	—
9. Sustained attention (*Score!* task)	8.20	1.94	−0.21	0.40**	−0.17*	0.50***	0.40**	0.22	−0.05	0.06

RT: response time.

* *p* < .05, **p < .01, ***p < .001.

### Discussion

The pilot study was designed to examine the effects of including self-pronouns on participants’ processing of mathematical problems. A large effect of referent on accuracy was found, with self-pronoun problems eliciting higher accuracy than problems without a self-pronoun. However, this pattern was complicated by an unexpected three-way interaction: self-pronouns produced an accuracy advantage in addition problems only when positioned first, whereas in the more difficult subtraction problems, there was a non-significant trend towards self-pronouns producing an advantage only when not positioned first. This runs contrary to D’Ailly et al.’s (1997) suggestion that later positioning of self-pronouns is disruptive in more difficult problems. There was no support for the proposal that including a self-pronoun reduces the working memory load of the problem simply by reducing the number of new characters to be held active during processing, as the self-advantage was not limited to conditions in which participants were attempting to track multiple characters. In terms of RT, an advantage for self-referenced problems emerged only in the addition condition. Overall, these pilot data suggest that self-referencing can have a positive effect on children’s mathematical problem solving, but that the effect may be conditional.

Interestingly, there were no consistent associations found between the accuracy or RT advantage for self-referenced problems and the attentional measures completed by participants, with only one test of sustained attention showing a positive relationship with the RT advantage. These measures covered working memory (forward digit span and BDS), sustained and selective attention to a task, and task switching. The lack of consistent relationships suggests that the facilitative effects on children’s ability to solve self-referent over other referent problems may not be strongly not linked to the measured aspects of attention.

In interpreting the pilot study, it should be noted that the experimental design was relatively complex for the number of trials. Although the study was sufficiently powered to detect the large effect of self-referencing on problem-solving accuracy, cell values in individual referent, operation, position, and tracking combinations were based on a small number of trials, so paired comparison results could have been driven by relatively few errors per condition, and small correlations may not have been detected. Having confirmed that the self-reference effect in numerical problem solving is replicable, it is therefore important to probe conditional effects further in our main study using a simplified design with more trials per condition.

## Main study

In the pilot study, difficulty was inferred from operation rather than being manipulated directly. This inference was supported by addition problems being solved more quickly and accurately than subtraction problems, but the interpretation is complicated by the confound between difficulty and operation. The main study was therefore designed to test the effects of self-pronoun inclusion on addition and subtraction problems with a specific difficulty manipulation based on wording consistency.

Consistency refers to the matching of relational words included in a problem with the operation required to solve that problem (see de Koning & van der Schoot, 2019). When the operation is addition, consistent problems include relational terms like “more than” (e.g., “*John has five apples, Sally has three apples more than John. How many apples does Sally have?*”), while inconsistent problems include phrases like “less than” (e.g., *“John has five apples, John has three apples less than Sally. How many apples does Sally have?*”). Subtraction problems include the opposite pairing, such as “less than” in consistent problems and “more than” in inconsistent problems. Research suggests that children find consistent problems much easier to solve than inconsistent problems, in both addition and subtraction (e.g., de Koning & van der Schoot, 2019; Hegarty et al., 1992; Lewis & Mayer, 1987; for a review, see Daroczy et al., 2015).

Interestingly, one previous study has examined the inclusion of self-pronouns in consistent and inconsistent word problems. de Koning and van der Schoot (2019) asked 9- and 10-year-old children to solve problems based on shopping tasks in which the store either did or did not belong to self (e.g., *“At Intertoys, a Lego box costs 31* *euros. That is 15* *euros less than at your store. How much will you pay at your store?*). This paradigm revealed no effects of self-reference, although a significant consistency effect was found with more consistent than inconsistent problems being answered accurately. However, the self-pronoun term (i.e., ‘your store”) was never the first word in the problem, and only appeared in the first sentence on one-third of self-referent trials. More importantly, while store owner was used to manipulate reference, the problem was always phrased in the second person (“*How much will you pay at [named store]?*,” emphasis added). This means that there was a self-pronoun included in each word problem whether it was in the self- or other referent condition. Together, these issues make it unsurprising that de Koning and van der Schoot (2019) found no overall effects of self-reference on accuracy, although the consistency manipulation was effective in creating two dissociable levels of difficulty.

Building on de Koning and van der Schoot’s approach by including clearly distinct self-referent and other referent conditions, in this study, we included a consistency manipulation in self- and other-referent word problems of the same format as those used in the pilot. We also tested a slightly older age range (9–12 years), to increase the likelihood that all children in the sample would be familiar with the different question type requirements. A short test of working memory capacity was included. While the pilot study did not show a relationship between the accuracy advantage for self-pronouns and any measure of attention, and there was only one measure (of sustained attention) linked with the RT advantage, there may be more scope to find a relationship with attentional capacity in the main study as it includes more difficult problems and a larger sample. It is well established that children who have poorer working memory tend to find mathematical processing more difficult than those with a higher capacity (Best et al., 2011; Bull & Scerif, 2001; Fuchs et al., 2005; Raghubar et al., 2010). Manipulations of difficulty may therefore vary in their effectiveness across children of different working memory capacities. The working memory task included in the main study was BDS, the most commonly used clinical and neuropsychological measure of working memory capacity (see Hilbert et al., 2015). BDS has been widely used to examine links between working memory and mathematical processing, revealing a significant relationship between the two (e.g., Bull et al., 2008; Hope & Sherrill, 1987; Ramirez et al., 2013). Here, we will examine whether there is any association between working memory capacity and any advantage for self-referential questions.

To avoid the issue of small trial numbers within each condition encountered in our pilot data, a larger sample was recruited, and other experimental factors were kept constant: the repeating character (self or other) was always positioned in the anchoring position, and the word problems all involved tracking two characters. The main hypothesis was that self-pronouns would enhance speed and accuracy on the problem-solving task. We also expected more difficult inconsistent problems to incur slower and less accurate responses than the easier consistent problems. Finally, if the use of self-referential processing reduces demands on working memory then it could be predicted that problems with self-pronouns would be most effective in the most difficult problems (i.e., subtraction and inconsistent word problems), where higher working memory demands are offset by the use of self-cues.

### Method

#### Participants and design

The sample comprised 144 children aged 9 to 12 years, with twenty-one 9 year-olds (42.86% male, age range = 11 months), forty-one 10 year-olds (43.9% male, range = 11 months), seventy 11 year-olds (55.7% male, age range = 11 months), and twelve 12 year-olds (66.7% male, range = 7 months). All participants were Primary 6–7 pupils at local primary schools and had no reported problems speaking or reading English. The children were tested with the online consent of a parent or guardian and the research was approved by Abertay University’s Research Ethics Committee.

The experiment had a repeated measures design of 2 (Referent: Self, Other) × 2 (Operation: Addition, Subtraction) × 2 (Problem consistency: Consistent, Inconsistent). Dependent measures were problem solving accuracy and RT. Participants’ BDS was also included as an exploratory measure. The study was pre-registered using the AsPredicted template with a sample size calculation based on a predicted medium effect size for referent (note, for pandemic-related practical reasons this pre-registration was completed prior to a full analysis of our pilot data). Based on a medium effect size (*η_p_*^2^ = .06) and a 2-level within-subjects effect of interest, G*Power 3.1.9.7. calculated an appropriate sample size to be 126 participants (α = .05, power = .80).

#### Materials and procedure

Children were tested in groups of four with two experimenters supervising each group. Testing took place when pupils returned to Scottish schools following a lengthy closure for the COVID-19 pandemic. The children sat at socially distanced desks in a quiet area of the school with a school laptop. Each testing session lasted around 30 min. For logistical reasons, the experimental administration platform was changed to Gorilla Online Experiment Builder (www.gorilla.sc), a cloud-based research platform used to deploy behavioural experiments online. The children were instructed to copy and paste the experimental URL link into a browser to begin the experiment, with experimenters present to facilitate progress through the tasks. The experimental programme delivered two tasks in the same order for each child: a mathematics problem-solving task and a BDS task.

##### Problem-solving task

Children were presented with instructions that asked them to read each question carefully (without reading aloud), to type their answer using the laptop keyboard and press “Next” using the mouse or touchpad to submit their response. They then completed four practice questions (addition consistent; addition inconsistent; subtraction consistent; subtraction inconsistent, with the order randomised between participants) to build familiarity with the type of problems in the task.

Participants completed three short blocks of self-paced trials, with the opportunity for a short break between each block. A total of 32 test questions were presented across three blocks (11 questions in Blocks 1 and 2, and 10 questions in Block 3), with four items per combination of manipulations. The order of the three blocks was randomised using Gorilla’s in-built randomiser tool, as was the order of the questions within each block. After each block children were presented with a rest screen with notes saying that they could take a break, as well as a button to continue the task. In addition, if children were unable to answer any question, the experimenter would prompt the child to “take a guess” or type “idk” (I don’t know) to move on to the next question.

Of the 32 problems included in the task, half were addition and half subtraction questions, as in the pilot (see Online Supplementary Materials for full list of word problems). Also, as in the pilot, half of the questions included the self-referent pronoun “You,” whereas this was replaced in the other half with a repeating three-letter Other-referent name which was the opposite gender to the child (Zak/Eve). However, to reduce the likelihood that the name used as the other-referent matched the participant’s own name, less common names than Sam were used. Specifically, Zak and Eve were chosen as they were the least common 3-letter names out of the 100 most popular names in the United Kingdom from the years in which the children were born (2009–2012). Children who chose “Other” or “Prefer not to say” as their gender (*n* = 3) were randomly assigned by Gorilla to either the male or female Other referent.

To manipulate question difficulty, the word consistency of the problem was manipulated such that half of the problems in each condition (i.e., Self v. Other, Addition v. subtraction) were presented in a Consistent format and half in an Inconsistent format. In Consistent questions, the term of the problem matched the operation required to solve it perform, so addition problems used the term “more” and subtractions used the term “less” (e.g., Addition: *‘You have 2 pillows. Candice has 4 pillows more than you. How many pillows does Candice have?’*; Subtraction: *“You have 9 biscuits. Murdo has 1 biscuit less than you. How many biscuits does Murdo have?”*). In contrast, in Inconsistent questions the term used in the problem does not match the operation required to solve it (e.g., Addition: *‘You have 2 pencils. You have 5 pencils less than Cara. How many pencils does Cara have?’*; Subtraction: *“You have 15 chocolate bars. You have 5 chocolate bars more than Ahmed. How many chocolate bars does Ahmed have?”*). All word problems were 17 words long. Word problem order was randomised within-participants using Gorilla’s spreadsheet randomiser tool. We also counterbalanced which half of the word problems was associated with each referent (Self vs Other) between participants (*n* = 68 for one half and *n* = 76 for the other).

##### BDS task

Following completion of all 32 questions in the problem-solving task, children were presented with a BDS task adapted from the Wechsler Intelligence Scale for Children - Fifth UK Edition (WISC-V UK) (Wechsler, 2016). A fixation cross appeared on a blank screen (Gorilla positions: 7 from top, 78 from bottom, full-screen width) for 1,000 ms and then disappeared. After a blank pause for 200 ms, children saw a series of digits appear in the same location as the cross. Each digit appeared for 1,000 ms and then was replaced by the next digit. After all the digits in a trial had been presented, a response box was shown with the following instructions: “Use the keyboard to type in the numbers in reverse order and press the Enter key when you’re done.”

Children first completed two 2-digit practice trials for the BDS tasks. If they typed an incorrect answer, they received a reminder reiterating the task instructions before being given the opportunity to repeat the practice trial. This process was repeated until they entered the correct answer. After successfully completing the first practice trial, the same process was repeated in a second practice trial. After completing this, children were moved on to the experimental trials of the BDS task.

The BDS task was divided into levels starting at 2 digits and going up to 7 digits per trial. At each level, there were two opportunities to pass a trial. If children gave the correct answer on one or both of the trials on a given level, they advanced to the next level. If they responded incorrectly for both trials of a level, the task ended.

### Data analysis

Participants’ performance on the problem-solving task was scored for accuracy (proportion of 32 problems correctly answered in each condition) and RT (latency from question onset to response submission on correct trials). All data were coded automatically by Gorilla, but experimenter checks revealed a small number of text responses that were manually corrected as they matched the correct answer but were not presented as integer responses (e.g., a text response of *“eleven”* or “*11 oranges*,” when the correct answer was “11”). Exclusions were also applied to the data, following the criteria applied in pilot testing. First, all trials below 200 ms (*n* = 3) were removed. Then we excluded children whose performance, in terms of overall accuracy in the task, was below the mean − 2.5 *SD* (*n* = 6), and children whose RTs were greater than the mean + 2.5 *SD* (*n* = 3). This meant that 135 children and 4317 trials were included in the analyses.

The plan for data analysis followed that of the pilot study exactly, with ANOVAs to examine accuracy and RT on the problem-solving task, and bivariate Pearson’s correlation to examine relations between the self-advantage score in accuracy and RT, and individual differences in age and working memory.

This study was pre-registered using the AsPredicted template, which can be accessed at https://doi.org/10.17605/OSF.IO/6FGHV. The full dataset and analysis scripts can be accessed at https://osf.io/naqz5/

### Results

#### Accuracy

Performance was high, with an overall accuracy of 89.4%, 95% CI [88.1%, 90.7%], and RT to correct trials 16.3 s, 95% CI [15.7s, 16.8s] per question. Accuracy scores were submitted to a 2 × 2 × 2 repeated measures ANOVA with Referent (Self vs Other), Operation (Addition vs Subtraction), and Consistency (Consistent vs Inconsistent) as the independent variables (see Online Supplementary Materials for full mean and standard deviations broken down by condition). As Table 4 shows, there was a main effect of Referent, with children correctly answering more Self-referent questions, *M* = 0.92, 95% CI [0.89, 0.94] than Other-referent questions, *M* = 0.87, 95% CI [0.85, 0.90]. There was also a main effect of Consistency, with children correctly answering more Consistent, *M* = 0.94, 95% CI [0.92, 0.97] than Inconsistent questions, *M* = 0.84, 95% CI [0.82, 0.87].

**Table 4.** ANOVA for main study accuracy (all df_num = 1, df_den = 134).

Predictor	*F*	*p*	*η*_p_^2^
Referent	25.39	<.001***	.159
Operation	0.00	.982	<.001
Consistency	49.47	<.001***	.270
Referent × Operation	10.51	.002**	.073
Referent × Consistency	12.35	<.001***	.084
Operation × Consistency	0.00	.952	<.001
Referent × Operation × Consistency	5.98	.016*	.043

* *p* < .05, **p < .01, ***p < .001.

**Figure 2.**
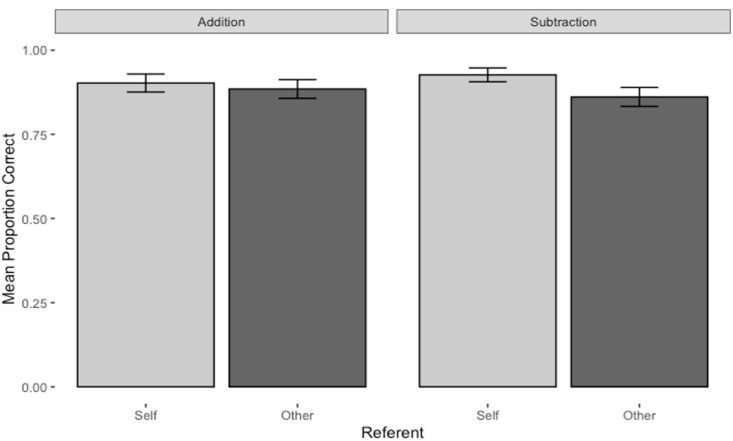
Mean proportion correct by referent for each operation in the main study. The error bars represent 95% confidence intervals.

The effect of Operation was not significant, but this was complicated by a significant interaction between Referent and Operation. Simple main effects for each level of Operation showed that the interaction arose because in Subtraction questions, accuracy was significantly higher under the Self-referent condition, *M* = 0.93, 95% CI [0.90, 0.95] than that under the Other referent condition, *M* = 0.86, 95% CI [0.83, 0.89], *F*(1, 134) = 32.3, *p* < .001, *η_p_*^2^ = .19, whereas in Addition questions, the two conditions did not differ significantly (self-referent *M* = 0.90, 95% CI [0.87, 0.93], other referent *M* = 0.88, 95% CI [0.85, 0.92], *F*(1, 134) = 2.79, *p* = .10, *η_p_*^2^ = .02; see Figure 2).

**Figure 3.**
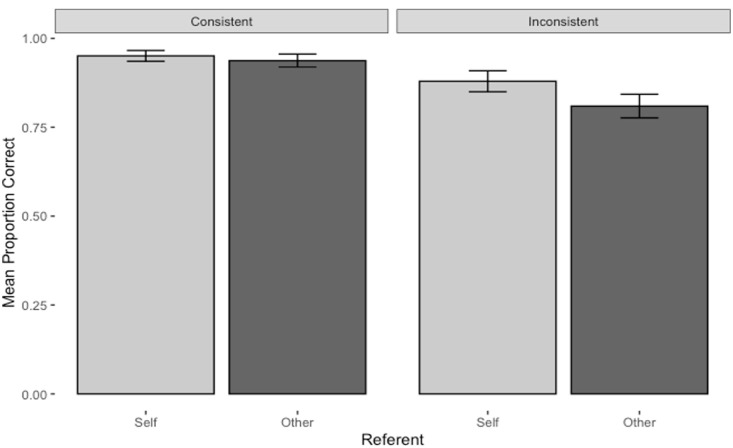
Mean proportion correct for self and other consistent and inconsistent questions in the main study. Error bars represent 95% confidence intervals.

There was also a significant interaction between Referent and Consistency. Simple main effects for each level of Consistency showed that there was an accuracy advantage for Self-referent questions under the more difficult Inconsistent condition (self-referent *M* = 0.88, 95% CI [0.84, 0.91], other-referent *M* = 0.81, 95% CI [0.77, 0.85], *F*(1, 134) = 28.0, *p* < .001, *η_p_*^2^ = .17) but not under the Consistent condition (self-referent *M* = 0.95, 95% CI [0.94, 0.97], other referent *M* = 0.94, 95% CI [0.92, 0.96], *F*(1, 134) = 1.95, *p* = .165, *η_p_*^2^ = .01; see Figure 3).

There was a three-way interaction between Referent, Operation and Consistency. Analysis of interaction effects for Referent × Operation at each level of Consistency showed that the interaction was not significant when the problems were Consistent, *F*(1, 134) = .05, *p* = .825, *η_p_*^2^ < .001. However, there was a significant Referent × Operation interaction when the problems were inconsistent, *F*(1, 134) = 12.2, *p* < .001, *η_p_*^2^ = .08. Simple main effects for the Inconsistent problems for each type of Operation showed accuracy was significantly higher for Self-referent questions, *M* = 0.90, 95% CI [0.87, 0.94] over Other-referent questions *M* = 0.79, 95% CI [0.74, 0.83] only for the most difficult Inconsistent Subtraction questions, *F*(1, 134) = 39.9, *p* *<* .001, *η_p_*^2^ = .23. There was no significant difference between self- and other referent conditions for the addition questions (self-referent *M* = 0.86, 95% CI [0.81, 0.90], other referent *M* = 0.83, 95% CI [0.78, 0.88], *F*(1, 134) = 1.65, *p* = .20, *η_p_*^2^ = .01).

#### Response time

We conducted the same 2 (Referent: Self, Other) × 2 (Operation: Addition, Subtraction) × 2 (Consistency: Consistent, Inconsistent) repeated measures ANOVA on participants’ mean RT to correct trials (see Table 5).

**Table 5.** ANOVA for main study response time (all df_num = 1, df_den = 134).

Predictor	*F*	*P*	*η*_p_^2^
Referent	31.95	<.001***	.193
Operation	1.46	.229	.011
Consistency	68.64	<.001***	.339
Referent × Operation	0.00	.995	<.001
Referent × Consistency	0.36	.550	.003
Operation × Consistency	1.46	.230	.011
Referent × Operation × Consistency	0.22	.638	.002

*** *p* < .001.

**Figure 4.**
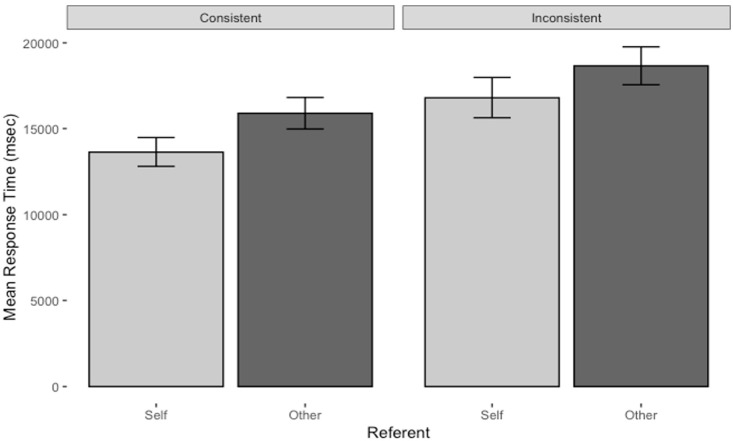
Mean response time for self and other consistent and inconsistent questions in the main study. Error bars represent 95% confidence intervals.

The ANOVA revealed a main effect of Referent, with Self questions being answered more quickly, *M* = 15.22 s, 95% CI [14.07, 16.37], than Other questions, *M* = 17.28 s, 95% CI [16.13, 18.43] (see Figure 4). There was also a main effect of Consistency, with Consistent questions being answered more quickly, *M* = 14.77 s, 95% CI [13.62, 15.92], than Inconsistent questions, *M* = 17.77 s, 95% CI [16.58, 18.88]. There was no main effect of Operation and no two- or three-way interactions reached significance.

##### Exploratory analysis: self-advantage associations

To examine relationships between the performance advantage for Self-referent over Other referent questions and the other variables of interest (total accuracy; BDS; age), an advantage score was calculated for both accuracy (performance in Self minus Other referent condition) and RT (performance in Other minus Self-referent condition) for each participant. BDS was scored by categorising participants by the highest level they reached on the BDS task—participants could range from 1 (“Failing Two Digits”) to 7 (“Passing Seven Digits”). As Table 6 shows, there were only two significant correlations. First, there was a negative relationship between participants’ total accuracy and the accuracy advantage for self over Other questions, *r*(133) = −.391, *p* < .001, suggesting that the more difficult the children found the mathematics task overall, the more of an advantage self-pronouns produced. Second, as would be expected there was a positive relationship between participants’ total accuracy and BDS, *r*(133) = .268, *p* = .002; children with higher digit spans tended to perform better on the arithmetic tasks overall.

**Table 6.** Correlations for the self-referent advantage in accuracy and response time, total accuracy, backward digit span, and age in months in the main study.

	*n*	*M*	*SD*	Pearson’s correlation coefficient				
				1	2	3	4	5
1. Self-advantage accuracy	135	0.041	0.095	—				
2. Self-advantage RT	135	2.05s	4.22s	.06	—			
3. Age in months	133	131.8	9.108	−.12	.09	—		
4. Total accuracy	135	0.894	0.136	−.39***	.09	.16	—	
5. Backward digit span	135	3.556	1.443	−.13	−.01	.14	.27**	—

RT: response time.

** *p* < .01, ***p < .001.

### Discussion

We tested the effect of including self-pronouns in numerical problems of different levels of difficulty, in a larger sample and with more items per condition than in our pilot. Results replicated the main pilot finding with self-pronouns significantly increasing accuracy, as well as RT being faster in these problems. The effect of self-pronouns on accuracy was stronger in the most difficult questions, with significant accuracy advantages for self-referent problems emerging in subtraction but not addition, and in inconsistent but not consistent questions. Accordingly, analyses of a three-way interaction showed that the accuracy advantage for self-referenced problems was driven by responses to the inconsistent subtraction questions.

The facilitative effect of including self-pronouns in word problems replicates the key finding of the pilot study, and supports D’Ailly and colleagues’ (1995, 1997) findings suggesting that self-referencing can play a role in supporting children’s numerical processing. Interestingly, whereas the mechanisms purported to underpin this relationship are attention-based, there was no relationship between the facilitative effect of self-pronouns and children’s age or working memory capacity. Although a lack of relationship needs to be interpreted cautiously, this pattern is consistent with the explanation that self-referent items are effective because they automatically attract attention and engage the child in the task, regardless of individual differences in processing capacity.

The stronger effects of self-referencing on problems with a higher difficulty was predicted because it was reasoned that self-referent cues should reduce working memory load, and therefore be of most benefit in the most difficult problems (i.e., subtraction and inconsistent word problems). However, this is in contrast with D’Ailly et al.’s (1997) finding that when positioned first (as they were in the current experiment), self-pronouns conferred an advantage across difficulty levels. Furthermore, in our pilot study, we found a significant facilitative effect in RT only in the easier addition problems. Here, we found the strongest advantage for the most difficult condition and like the pilot study, this pattern was also evident in the exploratory correlations with a significant negative relationship between the self-advantage for accuracy and total accuracy on the task. A potential explanation for this pattern is that ceiling effects may have reduced the ability of the experiment to detect self-other differences under easier conditions; for example, mean accuracy for other-referent consistent problems was 94% (*SD* 15%), leaving the limited potential for performance in the self-referent condition to reliably exceed this figure. Using more difficult questions overall (e.g., with double-digit integers) may have resulted in a similar effect of self-pronouns emerging across levels of difficulty as the RT facilitation provided by self-pronouns was strong across all conditions, with no interaction between referent and consistency.

## General discussion

The current study examined the effects of including the self-pronoun “you” on children’s solving of numerical word problems. Both the pilot and main experiments provided evidence for a medium to large beneficial effect of including self-pronouns on performance, although the strength of this effect varied across conditions and measures. In the pilot study, it was found that self-pronouns resulted in significantly faster responses only in addition problems and was somewhat inconsistent across operation and pronoun position combinations, although interpretation of these interactions in a small sample should be cautious. In the main study, when the self-pronoun was always positioned first, self-pronouns resulted in consistently faster responses across conditions, and increased accuracy, particularly in the more difficult subtraction and inconsistent problems.

The key finding of the study is that the inclusion of self-pronouns has a strong, facilitative effect on children’s problem-solving performance. This provides an important replication of D’Ailly et al.’s (1997) novel report, suggesting that self-referent manipulations would provide effective support for mathematical processing in an educational context. Children face many barriers while learning to solve mathematical problems, from reading comprehension and interpretation to mathematical understanding and arithmetic skills (see Boonen et al., 2016). Any manipulations that can be applied to minimise these barriers should benefit children’s acquisition of problem solving. As such, simple and effective measures like including self-pronouns in word problems are highly recommended.

The effects of self-cues on the attention system are clear and well established, suggesting these provide the most likely explanation for the current findings. While processing a task with a high working memory load such as a mathematical word problem, self-cues could provide support by automatically capturing attention and thereby reducing the working memory load of attention allocation, or by providing more effective storage (as a result of increased familiarity or organisation) and thus reducing the competition for attentional resources between storage and processing (Cowan, 2005). The precise interaction between self-cues and attention that supports problem-solving performance requires further empirical exploration, but these proposed mechanisms provide a plausible account of the facilitative effects reported in the current study.

Previous research on personalization provides some additional insight into the potential for self-cues to increase performance through task engagement, which is likely to motivate increased attention to the task (Høgheim & Reber, 2015; Reber et al., 2018). Personalization in learning tasks has occasionally been achieved using first-person perspective and personal pronouns, such as asking children to write sentences beginning with the word “I” (Turk et al., 2015), or including “you” in task instructions (Moreno & Mayer, 2000). This approach has demonstrated effects on task engagement, with children writing longer first- than third-person sentences for example (Turk et al., 2015). However, there are inconsistent effects of personalization on performance (e.g., Akinsola & Awofala, 2009; Bates & Wiest, 2004; Høgheim & Reber, 2015; Van de Weijer-Bergsma & Van der Ven, 2021) suggesting that it may not reliably activate the self-referential processing biases associated with self-cues. Furthermore, unlike personalization, using personal pronouns provides a tool that is applicable to every child, and the large effect sizes found in the current study suggest it may be a more effective and efficient technique, perhaps combining the task engagement associated with personalization with the more low-level attentional effects produced by self-referencing (Humphreys & Sui, 2016).

There was very little evidence of an association between the processing advantages elicited by self-cues, and individual differences in attentional processing or working memory capacity. While null findings need to be interpreted with caution, the lack of a consistent relationship between self-referential advantages and measures of attention and working memory capacity suggests that children with differing levels of attentional resource availability do not differ strongly in the extent to which they benefit from self-processing biases. This is consistent with other research in which self-reference effects do not correlate strongly with other individual differences in childhood cognition (e.g., Cunningham et al., 2013, 2014), speaking to their relatively automatic, universal nature. However, more research is required to determine whether the potential link with sustained attention is reliable, and whether children who are particularly low in working memory capacity (i.e., beyond the variance of the current sample) may experience more support for self-referent cues.

An additional important area for future research is the extent to which self-pronouns enhance accuracy and processing time across different problem types. Here, we found some contradictory patterns. In our pilot study, we found that the self-reference effect in RT was significant only in the easier, addition problems, although this pattern was based on a relatively small dataset. When difficulty was manipulated in the main study, we found a consistent self-reference advantage in RT across conditions. In the accuracy data, the study showed the self-reference effect was strongest under the most difficult (inconsistent, subtraction) conditions, a pattern that did not emerge in our pilot testing or D’Ailly et al.’s (1997) research. Although the main study pattern may have emerged as a result of self-cues facilitating performance particularly when working memory demands were high, an issue that may have affected the accuracy results is a ceiling effect potentially masking self-referential advantages under the easier conditions. An additional difference with D’Ailly et al.’s study is terms of mode of delivery; items in the current study were presented on-screen for the duration of the trials, as opposed to the verbal presentation used in D’Ailly and colleagues’ research. Time-unlimited on-screen presentation reduces the likelihood that any disrupting effects of self-cues will interfere with the processing of other information in difficult tasks, as missed information can simply be revisited on-screen in a way that is not possible with sequentially presented verbal information. Therefore, the effects of self-referent cues in tasks of levels of difficulty require further exploration.

Further research is also required to address the limitations of the current work in an educational context. In particular, more research is needed to elucidate how self-reference effects change depending on the type of problem. Here, we used numerical word problems adapted from types used in the local curriculum, to ensure our results could be applied to common school activities. However, these word problems include linguistic as well as numerical complexity, so the advantage for self-referenced problems found here may not generalise to other types of mathematical problems. Also, while it is clear that there are benefits of including self-pronouns in the immediate task of problem solving, this does not imply an improvement in long-term learning or skill acquisition. By reducing some of the attentional load of problem solving, it could be reasoned that children will have the cognitive capacity to more effectively transfer their numerical processing from practice to skills (see Paas, 1992; Renkl & Atkinson, 2003), but this prediction is as yet untested.

Overall, the current study suggests that there are reliable advantages associated with self-referencing in terms of both accuracy and processing time. Although more research is needed to elucidate how these effects interact with problem type and difficulty, it is clear that the immediate effects of including self-pronouns in mathematical problem solving are reliable and beneficial, and that these represent a useful tool to support children’s processing.

## Supplemental Material

sj-docx-1-qjp-10.1177_17470218231174229 – Supplemental material for Put you in the problem: Effects of self-pronouns on mathematical problem-solving Click here for additional data file. Supplemental material, sj-docx-1-qjp-10.1177_17470218231174229 for Put you in the problem: Effects of self-pronouns on mathematical problem-solving by Sheila J Cunningham, Zahra Ahmed, Joshua March, Karen Golden, Charlotte Wilks, Josephine Ross and Janet F McLean in Quarterly Journal of Experimental Psychology
