# Supplementary material for: Put you in the problem: Effects of self-pronouns on mathematical problem-solving
Source: Q J Exp Psychol (Hove). 2023 May 23;77(2):308–25. doi: 10.1177/17470218231174229 (PMC10798033; doi:10.1177/17470218231174229)
Supplement: sj-docx-1-qjp-10.1177_17470218231174229 – Supplemental material for Put you in the problem: Effects of self-pronouns on mathematical problem-solving [file sj-docx-1-qjp-10.1177_17470218231174229.docx]

Supplementary Material for:

**Put you in the problem:**

**Effects of self-pronouns on mathematical problem solving**

Cunningham, S.J.^a^, Ahmed, Z.^a^, March, J.^a^, Golden, K.^a^, Wilks, C.^a^, Ross, J.^b^, & McLean, J.F.^a^

**Affiliations:**

1. School of Applied Sciences, Kydd Building, Abertay University, Bell Street, Dundee, UK, DD1 1HG
2. Psychology, School of Humanities, Social Sciences and Law, Scrymgeour Building, University of, Dundee, Dundee UK, DD1 4HN

**Corresponding author email:** s.cunningham@abertay.ac.uk

**Co-author emails:** z.ahmed1600@abertay.ac.uk, joshua.march@strath.ac.uk, k.golden1800@abertay.ac.uk, Charlotte.Wilks1@nottingham.ac.uk, J.U.Ross@dundee.ac.uk, j.mclean@abertay.ac.uk

**Supplementary Material 1**

Full list of Pilot study questions in one example counterbalancing condition (female participant):

| **Block 1 (multiple referents)**  Beth brought 2 cakes to the party and you brought 9 more. How many cakes were brought to the party? |
| --- |
| Sam has 2 grapes and Rachel gave him 7 more. How many grapes does Sam have now? |
| Abbie had 9 pictures and she gave some to you. She then had 6 pictures, how many did she give to you? |
| You had 9 grapes but Zahra took 2. How many grapes do you have now? |
| Anna has 7 sweets and you have 6 sweets. How many sweets are there altogether? |
| Fiona has 11 cakes and Sam has 2 fewer cakes than Fiona. How many cakes does Sam have? |
| You have 4 apples and John has 9 apples. How many apples are there altogether? |
| You had 12 stickers and you gave some to Gareth. You then had 3 stickers, how many did you give away? |
| Sam had 13 apples. He gave some apples to Angus, and had 4 left. How many apples did he give away? |
| Rose bought 6 bags to the trip, and Sam bought 9 more. How many bags were bought for the trip? |
| You have 13 toys. Shaun has 8 fewer toys than you. How many does Shaun have? |
| Ross had 11 cartons but you took 8 of them. How many cartons does Ross have now? |
| Ethan had 12 stickers but Ethan took 8 of them. How many stickers does Ethan have now? |
| Sam had 10 pencils but Jessica took 8 of them. How many pencils does Sam have now? |
| Nathan has 8 cartoons and Sam gave him 3 more. How many cartoons does Nathan have now? |
| Catherine has 15 bags. You have 6 fewer bags than Catherine. How many bags do you have? |
| Sam brought 8 toys to school, and Joe brought 5 more. How many toys were brought altogether? |
| David has 8 stickers and you gave him 4 more stickers. How many stickers does David have now? |
| You have 8 pencils and Sarah gave you 2 more. How many pencils do you have now? |
| Ashleigh had 13 sweets. She gave some to you and then had 7 sweets left. How many sweets did she give away? |
| You brought 3 games to the sleepover and Chris brought 4. How many games were brought to the sleepover altogether? |
| Eva has 6 pictures and Sam has 3 pictures. How many pictures do they have altogether? |
| Sam has 3 stickers and Jack has 9 stickers. How many do they have altogether? |
| Sam has 7 games. Murray has 3 fewer games than Sam. How many games does Murray have? |
| **Block 2 (single referent)** |
| For the collection, you have 11 bags. You have 6 fewer boxes than bags. How many boxes do you have? |
| You had 8 stickers and cards, but gave 3 away. How many items did you have left? |
| Sam had 14 apples and bananas but gave 8 away. How many pieces of fruit did he have left? |
| At the competition, you won 6 stickers and 2 medals. How many prizes did you win altogether? |
| You have 8 apples and also have 6 bananas. How many pieces of fruit do you have altogether? |
| You had 9 fruits altogether, a mix of grapes and oranges. If 5 were grapes, how many were oranges? |
| At the competition, Sam won 8 stickers and medals. If 6 were stickers, how many were medals? |
| You have 11 toys. You have 4 fewer books than toys. How many books do you have? |
| For the collection, Sam brought 5 bags and 6 boxes. How many items did he bring altogether? |
| Altogether, Sam has 5 pictures and 9 puzzles. How many items does he have? |
| For the party, Sam bought 10 cakes. He bought 4 fewer cookies than cakes. How many cookies did he buy? |
| Sam took 4 toys and 7 books to the sleepover. How many things did he take to the sleepover altogether? |
| From the store, you bought 14 pictures and puzzles, but gave away 5. How many items did you have left? |
| You had 4 pencils, then you got 2 notepads. How many things do you have now? |
| From the bakery, Sam bought 10 sweets and doughnuts, but gave 3 away. How many did he have left? |
| Sam had 5 grapes then he was given 4 oranges. How many pieces of fruit does he have now? |
| Sam has 3 stickers and also has 5 cards. How many items does he have altogether? |
| You bought 5 games and 7 songs for the journey. How many things did you buy for the journey? |
| For drinks, you have a mix of 15 cartons and bottles. If 7 are cartons, how many are bottles? |
| First Sam had 7 cartons, then he was given 8 bottles. How many drinks does he have now? |
| Sam's 6-item stationery pack included both pencils and notepads. If 4 were pencils, how many were notepads? |
| For the party, you bought 6 cakes and 4 cookies. How many snacks did you buy altogether? |
| Sam has 12 games. He has 5 fewer songs than games. How many songs does he have? |
| Altogether you have 3 sweets and 7 doughnuts. How many snacks do you have? |

**Supplementary Material 2**

*Mean and Standard Deviation for the proportion of correct responses and response time by Referent, Operation, Tracking and Position in the Pilot study.*

| Referent | Operation | Tracking | Position | Proportion Correct | | Reaction Time (sec) | |
| --- | --- | --- | --- | --- | --- | --- | --- |
|  |  |  |  | Mean | SD | Mean | SD |
| Self | Addition | Single | Anchor | 0.98 | 0.08 | 12.71 | 5.29 |
|  |  |  | Not anchor | 0.93 | 0.15 | 12.85 | 6.93 |
|  |  | Multiple | Anchor | 0.94 | 0.15 | 12.35 | 5.86 |
|  |  |  | Not anchor | 0.90 | 0.15 | 13.88 | 8.15 |
|  | Subtraction | Single | Anchor | 0.85 | 0.22 | 19.69 | 9.41 |
|  |  |  | Not anchor | 0.84 | 0.25 | 19.74 | 10.32 |
|  |  | Multiple | Anchor | 0.85 | 0.22 | 17.79 | 9.69 |
|  |  |  | Not anchor | 0.87 | 0.21 | 19.01 | 9.23 |
| Other | Addition | Single | Anchor | 0.90 | 0.20 | 14.24 | 8.72 |
|  |  |  | Not anchor | 0.90 | 0.22 | 13.99 | 6.66 |
|  |  | Multiple | Anchor | 0.91 | 0.17 | 13.65 | 7.25 |
|  |  |  | Not anchor | 0.91 | 0.15 | 13.99 | 5.51 |
|  | Subtraction | Single | Anchor | 0.85 | 0.24 | 18.07 | 8.70 |
|  |  |  | Not anchor | 0.72 | 0.34 | 19.19 | 11.71 |
|  |  | Multiple | Anchor | 0.84 | 0.25 | 17.24 | 8.47 |
|  |  |  | Not anchor | 0.87 | 0.23 | 18.29 | 10.30 |

**Supplementary Material 3**

Table 1 shows that there was a significant interaction between Operation and Tracking for accuracy. Pairwise comparisons revealed that with subtraction problems participants were more accurate for with multiple referents (M = 0.86, 95% CI [0.81, 0.90]), than single referent (M = 0.81, 95% CI [0.76, 0.87]); t(49) = -2.21, p = .032. There was no difference for addition problems, single referent (M = 0.93, 95% CI [0.89, 0.96]) and multiple referents (M = 0.82, 95% CI [0.89, 0.94]); t(49) = 0.75, p = .455. Table 2 shows this interaction was also found with the response times. The pairwise comparisons revealed that for subtraction problems, participants were faster to respond to multiple referents (M = 18.1, 95% CI [15.8, 20.4]), than single referent (M = 19.2, 95% CI [16.6, 21.7]); but this difference was not significant t(49) = 1.64, p = .11. There was also no difference for addition problems, single referent (M = 13.4, 95% CI [11.8, 15.1]) and multiple referents (M = 13.5, 95% CI [11.9, 15.1]); t(49) = -0.04, p = .967.

For the accuracy data, there was also a significant interaction between Tacking and Position. Pairwise comparisons revealed that when the referent was not in anchoring position, participants were more accurate for with multiple referents (M = 0.89, 95% CI [0.86, 0.92]), than single referent (M = 0.85, 95% CI [0.80, 0.90]); t(49) = -2.04, p = .047. There was no difference when the referent was in an anchoring position, single referent (M = 0.89, 95% CI [0.86, 0.93]) and multiple referents (M = 0.89, 95% CI [0.85, 0.92]); t(49) = 0.52, p = .605.

**Supplementary Material 4**

Full list of main study questions in one example counterbalancing condition (male participant):

| **Block 1**  You have 2 pencils. You have 5 pencils less than Cara. How many pencils does Cara have? |
| --- |
| You have 4 marbles. Craig has 8 marbles more than you. How many marbles does Craig have? |
| You have 8 grapes. Kelly has 2 grapes less than you. How many grapes does Kelly have? |
| You have 14 bananas. You have 3 bananas more than Nathan. How many bananas does Nathan have? |
| Eve has 12 napkins. Megan has 7 napkins less than Eve. How many napkins does Megan have? |
| Eve has 2 pillows. Candice has 4 pillows more than Eve. How many pillows does Candice have? |
| Eve has 15 sweets. Sarah has 3 sweets less than Eve. How many sweets does Sarah have? |
| Eve has 5 hats. Eve has 6 hats less than Jacob. How many hats does Jacob have? |
| Eve has 11 games. Nicola has 6 games less than Eve. How many games does Nicola have? |
| You have 9 yoghurts. You have 5 yoghurts less than Ryan. How many yoghurts does Ryan have? |
| **Block 2** |
| You have 4 pots. Douglas has 11 pots more than you. How many pots does Douglas have? |
| You have 12 blocks. You have 3 blocks more than Zara. How many blocks does Zara have? |
| You have 10 bags. Manuel has 6 bags less than you. How many bags does Manuel have? |
| You have 11 tickets. You have 3 tickets more than Michael. How many tickets does Michael have? |
| You have 2 chocolate bars. Cathy has 11 chocolate bars more than you. How many chocolate bars does Cathy have? |
| You have 12 cakes. Maggie has 3 cakes more than you. How many cakes does Maggie have? |
| Eve has 15 chocolate bars. Eve has 5 chocolate bars more than Ahmed. How many chocolate bars does Ahmed have? |
| Eve has 3 rolls. Eve has 8 rolls less than Mark. How many rolls does Mark have? |
| Eve has 5 stickers. Eve has 8 stickers less than Stephanie. How many stickers does Stephanie have? |
| Eve has 6 marbles. Eve has 2 marbles more than Rose. How many marbles does Rose have? |
| Eve has 2 apples. Chetna has 3 apples more than Eve. How many apples does Chetna have? |
| **Block 3** |
| Eve has 4 toys. Lynda has 8 toys more than Eve. How many toys does Lynda have? |
| You have 14 oranges. You have 9 oranges more than Ethan. How many oranges does Ethan have? |
| You have 1 sticker. You have 7 stickers less than Keith. How many stickers does Keith have? |
| You have 7 flyers. Helen has 4 flyers less than you. How many flyers does Helen have? |
| You have 9 pencils. Ben has 4 pencils less than you. How many pencils does Ben have? |
| Eve has 9 biscuits. Murdo has 1 less biscuit than Eve. How many biscuits does Murdo have? |
| Eve has 6 forks. Maya has 2 forks more than Eve. How many forks does Maya have? |
| Eve has 14 grapes. Eve has 6 grapes more than Stephen. How many grapes does Stephen have? |
| You have 7 sweets. You have 3 sweets less than Joe. How many sweets does Joe have? |
| Eve has 9 apples. Eve has 6 apples more than Calum. How many apples does Calum have? |
| Eve has 8 cups. Eve has 7 cups less than Marvin. How many cups does Marvin have? |

**Supplementary Material 5**

*Mean and Standard Deviation for the proportion of correct responses and response time by Operation, Referent, and consistency in main study*

| Operation | Referent | Consistency | Proportion Correct | | Reaction Time (ms) | |
| --- | --- | --- | --- | --- | --- | --- |
|  |  |  | Mean | SD | Mean | SD |
| Self | Addition | Consistent | 0.95 | 0.13 | 12557 | 7167 |
|  |  | Inconsistent | 0.86 | 0.28 | 11963 | 5462 |
|  | Subtraction | Consistent | 0.95 | 0.13 | 17108 | 9384 |
|  |  | Inconsistent | 0.90 | 0.20 | 16031 | 7660 |
| Other | Addition | Consistent | 0.94 | 0.14 | 11841 | 4768 |
|  |  | Inconsistent | 0.83 | 0.28 | 11661 | 6028 |
|  | Subtraction | Consistent | 0.94 | 0.16 | 15840 | 7411 |
|  |  | Inconsistent | 0.79 | 0.27 | 16632 | 8258 |
